# Soil Selenium (Se) Biofortification Changes the Physiological, Biochemical and Epigenetic Responses to Water Stress in *Zea mays* L. by Inducing a Higher Drought Tolerance

**DOI:** 10.3389/fpls.2018.00389

**Published:** 2018-03-27

**Authors:** Marika Bocchini, Roberto D’Amato, Simona Ciancaleoni, Maria C. Fontanella, Carlo A. Palmerini, Gian M. Beone, Andrea Onofri, Valeria Negri, Gianpiero Marconi, Emidio Albertini, Daniela Businelli

**Affiliations:** ^1^Department of Agricultural, Food and Environmental Sciences, University of Perugia, Perugia, Italy; ^2^Department for Sustainable Food Process, Catholic University of the Sacred Heart, Piacenza, Italy

**Keywords:** selenium, maize, selenium speciation, water stress, DNA methylation, epigenetic

## Abstract

Requiring water and minerals to grow and to develop its organs, Maize (*Zea mays* L.) production and distribution is highly rainfall-dependent. Current global climatic changes reveal irregular rainfall patterns and this could represent for maize a stressing condition resulting in yield and productivity loss around the world. It is well known that low water availability leads the plant to adopt a number of metabolic alterations to overcome stress or reduce its effects. In this regard, selenium (Se), a trace element, can help reduce water damage caused by the overproduction of reactive oxygen species (ROS). Here we report the effects of exogenous Se supply on physiological and biochemical processes that may influence yield and quality of maize under drought stress conditions. Plants were grown in soil fertilized by adding 150 mg of Se (sodium selenite). We verified the effects of drought stress and Se treatment. Selenium biofortification proved more beneficial for maize plants when supplied at higher Se concentrations. The increase in proline, K concentrations and nitrogen metabolism in aerial parts of plants grown in Se-rich substrates, seems to prove that Se-biofortification increased plant resistance to water shortage conditions. Moreover, the increase of SeMeSeCys and SeCys2 forms in roots and aerial parts of Se-treated plants suggest resistance strategies to Se similar to those existing in Se-hyperaccumulator species. In addition, epigenetic changes in DNA methylation due to water stress and Se treatment were also investigated using methylation sensitive amplified polymorphism (MSAP). Results suggest that Se may be an activator of particular classes of genes that are involved in tolerance to abiotic stresses. In particular, PSY (phytoene synthase) gene, essential for maintaining leaf carotenoid contents, SDH (sorbitol dehydrogenase), whose activity regulates the level of important osmolytes during drought stress and ADH (alcohol dehydrogenase), whose activity plays a central role in biochemical adaptation to environmental stress. In conclusion, Se-biofortification could help maize plants to cope with drought stress conditions, by inducing a higher drought tolerance.

## Introduction

The use of water in agriculture is unevenly spread with a peak in summer when water is least available, maximizing detrimental impacts (Sharpe, 2009). More than 90% of agricultural area in the EU is rain fed, so crop water stress often underlies the inter-annual variability observed in grain production (EUROSTAT, 2010).

Maize (*Zea mays* L.) is one of the most important food crops in the world (it is produced on nearly 100 million hectares) and, along with rice and wheat, provides at least 30% of food calories to more than 4.5 billion people in 94 countries. It is one of the most important cereals both for human and animal consumption. Furthermore, maize is an important candidate crop for ethanol production. Maize is a major summer crop in the irrigated areas of the Mediterranean region. It is a water demanding crop that can reach high grain yields (10–12 t ha^-1^) only when water and nutrients are not limited (Pandey et al., 2000; Çakir, 2004). Water demand for maize is concentrated in the summer months when availability is lowest. Access to an adequate water supply, including more efficient use of all available water, is therefore critical to achieving improved yields.

Drought stress is one of the major environmental factors that inhibits many metabolic processes and consecutively slows down the development of the plant with loss of yield and productivity around the world. The arid and semi-arid regions of the globe, especially in developing countries, are at great risk because they are facing acute shortage of water.

Plants respond to environmental stress by alteration of the pattern of protein synthesis. These stress-induced proteins are considered to enhance the plants’ ability to survive the conditions of stress. The limited or non-availability of water reduces growth of crop plants through the effects on various physiological and biochemical processes.

Selenium had long been considered to be a toxic element for higher organisms, but in Schwarz and Foltz (1957) reported that low concentrations of Se are essential for dietary intake and interchangeable with vitamin E. Later it was discovered that Se mitigating environmental stress protects the cells of rats against intracellular oxidative damage (Rotruck et al., 1973). Since then, owing to its physiological and toxicological importance, Se has become an element of interest which has been investigated in humans, animals and in plants.

Selenium toxicity (400 μg kg^-1^) in humans and animals causes disruption of the digestive and nervous systems but, on the other hand, deficiency leads to free radical damage, such as greater risk of tumour formation, hypertension and atherosclerosis. Cereals are the major source of Se among plants (50–60 μg kg^-1^). Se content of food is highly dependent on soil Se bioavailability and the ability of plants to take up and accumulate Se in edible tissues (Bañuelos et al., 2017). In the soil, selenite is less bioavailable to plants than selenate, because the former is preferentially absorbed by iron oxides and/or hydroxides. Owing to this behavior in the soil, selenite is preferable to selenate; it is not dispersed with rainwater or irrigation, thus also minimizing the risks of pollution of the groundwater (Zhu et al., 2009).

Selenium is not considered essential for plant growth, but recent studies indicate its physiological and anti-oxidant properties play beneficial roles in plants exposed to various abiotic stress (Cartes et al., 2010). Se improves plant tolerance to DS by regulating water status (Yao et al., 2009), increasing chlorophyll in plant leaves (Dong et al., 2013), protecting against cadmium toxicity (Elkahoui et al., 2004), reducing damage caused by UV-radiation oxidative stress (Valkama et al., 2003) and enhancing chlorophyll content under light stress (Seppänen et al., 2003).

Selenium may also protect plants from fungal infection and invertebrate phloem-feeders (Hanson et al., 2004). Moreover, it has also been reported to improve the yield of food crops like wheat (Broadley et al., 2010), barley (Ducsay et al., 2009), rice (Wang et al., 2013), and maize (Chilimba et al., 2012).

Abiotic stresses such as low and high temperature (Akladious, 2012; Balal et al., 2016), salinity (Abul-Soud and Abd-Elrahman, 2016), heavy metals (Li et al., 2016), excess water and drought (Nawaz et al., 2015) could be regulated by exogenous Se, associated with the increased activity of GPX and antioxidants activity with a simultaneous decrease in lipid peroxidase activity. In Proietti et al. (2013), the effects of Se on plant growth under DS stimulate the activity of the antioxidant enzymes CAT and SOD, which were depressed in response to drought.

For example, an increase in antioxidant enzyme activity after Cd treatment has been detected in the roots and leaves of *Phaseolus vulgaris* L. (Chaoui et al., 1997) and *Raphanus sativus* L. (Vitória et al., 2001), such as the accumulation of H_2_O_2_, causing oxidative stress in these plants. DNA methylation play important roles in the establishment of developmental programs in fungi, plants and animals. (Teyssier et al., 2008). Environmental stimuli, such as heavy metals, water and salt stress, are known to alter cytosine methylation throughout the genome and specific loci (Lukens and Zhan, 2007). Plants use epigenetic regulatory strategies, such as DNA methylation, to maintain genomic plasticity, allowing relatively rapid adaptation to new conditions without changing the DNA sequence (Causevic et al., 2005). Generally, hypermethylation is correlated with gene silencing, while hypomethylation is connected with active transcription (Paszkowski and Whitham, 2001). Moreover, some authors suggest a role played by selenium in preventing DNA methylation changes and in reducing the amount of ROS, that, in turn, are believed to be the trigger of methylation status changes (Filek et al., 2008; Peng and Zhang, 2009). Therefore, in this paper, the possible involvement of epigenetic changes in maize in response to DS and/or Se supplementation was investigated through MSAP. This technique has been applied in several species to detect the methylation status and is based on the use of two isoschizomers, *Hpa*II and *Msp*I, recognizing 5′-CCGG-3′ sequences but have differential sensitivity to cytosine methylation status.

The aims of the work were: (i) to confirm the effectivity of the Se-biofortification technique used, which was able to transfer Se from soil to roots and aerial parts; (ii) to verify if Se-biofortification can increase the drought tolerance of maize plants and which mechanisms would be involved in this water stress tolerance, studying specifically the role of pigments, proteins, ions and DNA methylation.

## Materials and Methods

### Type of Soil Used

A clay-loam soil was air-dried, passed through a 2 mm sieve and analyzed for its basic physical and chemical properties. Water-holding capacity was 300 mL kg^-1^ and the permanent wilting point was 120 mL kg^-1^. On a dry weight basis: a pH (H_2_O) of 8.3, CaCO_3_ concentration of 240 g kg^-1^, total organic C of 9.9 g kg^-1^, total N concentration of 1.0 g kg^-1^, CSC of 20.1 cmol^(+)^ kg^-1^ and total Se concentration of 12.0 mg kg^-1^. Soil analyses were performed following official methods (Carter and Gregorich, 2007). For the determination of Se concentration, acid digestion of soil [0.25 g was performed with a mixture of HNO_3_ and H_2_O_2_ (8:2, v/v)]. The determination of the Se in digested materials was accomplished by using an atomic absorption spectrophotometer (US Environmental Protection Agency [EPA], 1996), equipped with a graphite furnace and a deuterium lamp (Shimadzu AA-6800, GF-AAS, “Shimadzu Corp.”, Tokyo, Japan). The back-ground correction was carried out using a matrix modifier of Pd(NO_3_)_2_ in 0.5 M in HNO_3_ (Sigma-Aldrich, St. Louis, MO, United States).

### Soil Preparation

Soil spiking was performed by taking samples of the same air dried and sieved (<2 mm) soil. The soil (3 kg) in the Se treated samples has been fertilized by adding 150 mg of Se (328 mg of sodium selenite) to each pot before sowing. The Se salt was added to the soil and then stirred for 4 h.

### Bioassay

The bioassay was carried out at the greenhouse of the Department of Agricultural, Food and Environmental Sciences, University of Perugia (Central Italy, 12°23-E, 43°5-N), in May 2015, with minimum temperature ranging between 10°C and 15°C and maximum temperature ranging from 20°C to 36°C.

The experimental design was completely randomized with four treatment factors: normal irrigation without Se treatment (WW-Se), DS without Se treatment (DS-Se), normal irrigation with Se treatment (WW+Se) and Se under DS condition (DS+Se) and six replicates, for a total of 24 pots. Maize (cv Fidias) was sown (11 May 2015) in 0.20 m diameter PVC pots filled with 3,000 g of air-dried soil, using three seeds per pot. For the samples under normal irrigation, the pots were sub-irrigated every 2nd day to recover water-holding capacity. For the stressed samples, DS was imposed on 5 June 2015, on plants 0.25 m high. From this date, irrigations were suspended to let the soil reach a water content equal to 25% of the available water. Later, the pots were sub irrigated every 2nd day to recover a water content equal to 25% of the available moisture content. The amount of water needed to reach 100 and 25%, respectively, of available moisture content in the soil were determined by considering the average weight of the pots and giving the same amount of water to all pots in the same irrigation treatment. Once in a week the sub-irrigations were performed by using a solution of fertilizer for hydroponics (FLORY 9, Agrochimica, Italy), containing 5% total N, 7% P_2_O_5_, 22% K_2_O, 6% MgO and less than 10 μg Se kg^-1^. Maize plants were grown up until pot size represented an obstacle to normal development; in detail, they were harvested at 0.50 m height (14 July 2015). After harvesting, all plants were rinsed quickly in deionized water, weighed and split into the different organs: roots and aerial parts (aerial parts and leaves).

### Plant Analyses

Plant aerial part height was determined by placing samples on black chart paper with a 1 m ruler alongside them. Portions of roots and aerial parts harvested were weighed and immediately used for the analysis that required fresh material (Se speciation and proline). Aerial part dry weight was obtained by drying samples in a forced-draft oven at 60°C for 48 h and ground to pass through a 0.5-mm sieve. Other portions were collected and rapidly frozen at -80°C and used for DNA extraction (MSAP technique).

### Total Chlorophylls and Carotenoids

The concentration of chlorophylls (Chlorophyll a, ChlA; Chlorophyll b, ChlB) and carotenoids (Car) were determined only in the fresh aerial part sample following the method of Lichtenthaler and Wellburn (1983).

The sum of ChlA and ChlB was reported as the total concentration of chlorophylls (TotChl). Results were then expressed as milligrams per gram of fresh weight (mg g^-1^ FW).

### Determination of Total Se Concentration

Dry vegetable samples (0.25 g) with additions of 8 mL of concentrated HNO_3_ (65% v/v; Carlo Erba, Milan, Italy) and 2 mL of H_2_O_2_ (30% v/v, Carlo Erba) were microwave digested (ETHOS One high-performance microwave digestion system; Milestone Inc., Sorisole, Bergamo, Italy) (Cubadda et al., 2010). The digested solutions were filtered using a 0.22 μm filter after appropriate dilution with Milli-Water (18.2 MΩ), according to US Environmental Protection Agency [EPA] (1996), and the concentration of total Se was determined by ICP-MS (Agilent 7900, Agilent Technologies, United States) with Octopole Reaction System (ORS system). The standard solutions of Se were used by diluting the corresponding stock solutions (Se standard 1000 mg L^-1^ for AAS TraceCert Sigma-Aldrich 89498). This method was accurately validated with a recovery test (*n* = 3) by adding from a Se standard solution (4 mg L^-1^) into the mixture of Se-enriched sample and nitric acid prior to digestion.

### Analysis of Se Species in Leaves and Roots

Approximately 1 g of fresh leaves and roots were accurately weighed and chopped with 10 mL of solution 2.0 mg mL^-1^ of protease (Protease Type XIV, Sigma-Aldrich P5147 -1 g). An ultrasound probe was used and sonication time was 3 min. All of the samples were stirred in a water bath at 37°C for 3 h. After the extraction, the samples were allowed to cool to room temperature and centrifuged for 10 min at 9,000 rpm. The supernatant was filtered through 0.22 μm Millex GV filters (Millipore Corporation, Billerica, MA, United States).

The Se standards (Na_2_SeO_3_, Na_2_SeO_4_, selenomethionine (SeMet), selenocystine (SeCys2), selenium methylselenocysteine (SeMeSeCys) were obtained from Sigma (St. Louis, MO, United States) and prepared in ultrapure (>18 MΩ) water.

Speciation of Se was performed by HPLC-ICP-MS (HPLC 1100 coupled with ICP-MS 7700x, both Agilent Technologies, United States) on an anion exchange column (Hamilton, PRP-X100, 250 × 4.6 mm, 5 μm particle size). The mobile phase was made using ammonium acetate (Carlo Erba, Milan, Italy) with gradient elution. Detailed information is summarized in **Supplementary Table S1**. The samples were analyzed at different dilutions and selenocompounds were identified in extracts by retention time matching with the standard substances spiked in the sample extracts.

Limit of detection (LODs = 3σ), expressed as μg L^-1^, were 0.9, 0.7, 0.3, 0.6, and 0.2 for SeCys2, MeSeCys, SeMet, Se(IV) and Se(VI), respectively.

### Proline Concentration

The concentration of proline was estimated in all plants by HPLC with a method described by Palmerini et al. (2006). The HPLC used was a Jasco 880-PU (Jasco, Tokyo, Japan) equipped with a fluorimetric detector Jascho 821-FP. The proline was measured on leaves (2 g of leaves) homogenized in 10 mL of ultrapure H_2_O with an ultra turrax T25 (Tanke and Kunkel Ika Labortechnik, Staufen, Germany) for 3 min on ice. The homogenates were then centrifuged at 5,000 × *g* for 10 min and an aliquot of the supernatant (1 mL) of the extract was deproteinized with 0.200 mL HClO_4_ (20% v/v) in ice and centrifuged at 8,000 rpm 5 min. At the supernatant were added 0.200 mL of KOH (20% by weight) and the precipitate of KClO_4_ was removed by centrifugation at 8,000 for 5 min. An aliquot of the supernatant (0.050 mL) was mixed with 0.150 mL (0.4 M) borate pH 9 with 0.050 mL of *o*-ftaldeide chloride (150 mM) (OPA) in methanol, and 0.1 mL of 7-chloro-4 -nitrobenzo 2 bone-1,3-diazole (25 mM) (NBD-Cl) in methanol. The reaction performed at 60°C for 3 min was interrupted in ice with 0.100 mL HCl (1 M). The derivatized (0.020 mL) of each sample was injected into a RP-18 Lichrosor column, (15 cm × 4.6 mm ID) of the HPLC and eluted in instrumentation under isocratic conditions with H_2_O/CH_3_CN (93:7) used as mobile phase. The solvents used were previously passed on a 0.22 μm filter (Millipore Corporation, Billerica, MA, United States). The NBD-derivatives were determined to ex_470 nm_ e em _530 nm_. The NBD- proline was eluted in 6.5 min and quantified with a standard solution of proline. Proline (0.043 M) and hydroxy-proline (0.038 M), used as internal standard, were diluted 1–100 in H_2_O and then 0.050 mL derivatized as the samples (0.01 mL) analyzed by HPLC. The results were expressed for proline as nmol g^-1^ TF.

### Ionic Concentrations in Aerial Parts

Ionic concentrations in plant extracts was determined by ion chromatography with conductivity detection (Portlab Hplc System Stayer, Milan, Italy). The method used was described for the determination of the ionic concentration of vegetable samples by ion chromatography with suppressed conductivity detection (Cataldi et al., 2003). Leaf tissues, approximately 0.25 g of (-Se) and (+Se) plants were homogenized in 5 mL of pure water; the suspension was shaken for 20 min and then centrifuged at 3,000 rpm for 12 min. Prior to the injection, the extracts were filtered through single-use 0.22 μm nylon filters to remove any particulate matter. The extracts obtained at room temperature yielded chromatographic profiles with substantial differences in the relative concentrations of F^-^, Cl^-^, Br^-^, NO_3_^-^, PO_4_^3-^, and SO_4_^2-^ as well as of Na^+^, K^+^. The results stated in this paper represent the mean of three repetitions performed for each sample. The concentrations of anions were both calculated as g 100 g^-1^, while for cations as mg g^-1^.

### Determination of Total Nitrogen (Kjeldahl Method)

Two grams of dry sample were digested in a Kjeldahl digestion flask, as in the official method used for the determination of total nitrogen in plant tissue (Isaac and Johnson, 1976) and which led to the use of the calculation N × 6.25 to convert nitrogen concentration into protein concentration (Nprot). Determinations were made on all reagents alone (blank determinations).

### DNA Extraction and MSAP Technique

For MSAP analysis, total genomic DNA, extracted from maize leaves, collected at 60 DAS using the DNeasy Plant Mini Kit (Qiagen, Milan, Italy), following the protocol reported in Marconi et al. (2013) and Bocchini et al. (2015). The 12 primer combinations used for the selective amplifications are reported in **Supplementary Table S2**. The differential sensitivity of *Hpa*II and *Msp*I to methylation allows the separation of the amplified fragments into four types. Type I is given by bands present in both enzyme combinations (*Eco*RI*/Hpa*II and *Eco*RI*/Msp*I). Type II correspond to bands present only in *Eco*RI*/Hpa*II, indicating the hemimethylated state of DNA, i.e., the result of the methylation in one strand but not in its complementary strand. Type III bands appeared only in *Eco*RI*/Msp*I, representing the case of full CG methylation (internal cytosine) whereas type IV, is the case of full methylation at both cytosines, and represents the absence of bands in both enzyme combinations.

### Statistical Analysis

Bioassay data relating to the concentrations of the different forms of Se in maize roots and aerial parts were submitted to two-way ANOVA, by considering water-stress and Se-biofortification as factors, with two levels each. The ‘water-stress × Se-biofortification’ interaction was always significant and the means for the four combinations were compared using Fisher’s LSD at *P* = 0.05. In order to summarize the results, principal component analysis (PCA) was used on the two-way matrix with treatments along the rows and all the observed variables along the columns [concentrations in sodium (Na) potassium (K), proteic nitrogen (Nprot), chlorophyll A (ChlA), chlorophyll B (ChlB), total chlorophyll (TotChl), carotenoids (Car), nitrates (Nitr), fluorides (Fluo), sulphates (Sulph), phosphates (Phosph) and chlorides (Chlor)]. Data were standardized prior to analysis and results were displayed in a distance-biplot (Legendre and Legendre, 2012).

In MSAP analysis, to determine the significance of the sources of variation, the recorded data (bands) were processed by analysis of variance (ANOVA). First, for each Type of methylation status (Types I, II, III, and IV), several one factor completely randomized ANOVAs were performed considering the stress treatments (i.e., WW+Se vs. WW-Se; WW+Se vs. DS+Se; WW+Se vs. DS-Se; WW-Se vs. DS+Se; WW-Se vs. DS-Se and DS+Se vs. DS-Se) as the experimental factor. The same simple one factor ANOVA was also carried out for the total methylated bands (i.e., the sum of bands of Types II, III, and IV), the full methylated bands (i.e., the sum of Types III and IV) and hemi methylated bands (Type II).

Secondly, a two-factor ANOVA with 6 replicates was performed, by considering Stress and Class (Total, Full and Hemi Methylated bands) as factors. In addition, the significance of effects was tested by using *F* tests. Pairwise comparisons were tested for the stress × class combinations. Finally, the mean ± standard deviation (SD) derived from six biological replicates per experiment was calculated for each MSAP band type.

### Silver Staining and DNA Sequences of Water-Selenium-Related Fragments

Some samples, which were chosen on the basis of interesting polymorphisms, were run on acrylamide gels and silver stained with the aim of isolating and sequencing the selected bands as reported in Marconi et al. (2013). Interesting polymorphic bands were then excised from gels and rehydrated with 100 μL of milli-Q water o/n at 4°C. Tubes were centrifuged at maximum speed for 5 min and the supernatant transferred into a fresh tube. Aliquots of 5 μL were used as a template for re-amplification by PCR in a 25 μL reaction volume. All PCR reactions were carried out with the same *Eco* and *Mps*I*/Hpa*II primer combination used in selective amplification step with this profile: 94°C for 1 min, 30 cycles of denaturation at 94°C for 1 min, annealing at 55°C for 1 min, extension at 72°C for 1 min and ending with a 20 min extension step at 72°C.

One microliter of the re-amplified DNA was cloned into pCR4-TOPO vector using the TOPO TA cloning kit for sequencing (Invitrogen, Carlsbad, CA, United States). Three plasmids for each transformation were purified from 5 mL of o/n culture of *E. coli*, in LB medium, using the GenElute Plasmid miniprep kit (Sigma-Aldrich, Milan, Italy). The sequences of both strands were determined after running sequencing reactions, obtained with BigDye^®^ Terminator v3.1 Cycle Sequencing Kit, (Applied Biosystems, Foster City, CA, United States) on an ABI 3130xl Genetic Analyzer (Applied Biosystems, Foster City, CA, United States). Sequences identified through MSAP were used as queries in NCBI Release 215^1^, Uniprot release 2016_08^2^ (The UniProt Consortium, 2017) and MaizeGDB^3^ by using B73 RefGen_v3 websites.

## Results

### Effects of Se-Biofortification on Plant Morphological, Chemical and Biochemical Parameters

In untreated (-Se) plants, DS significantly reduced aerial part weight by 37% (**Table 1** and **Supplementary Table S3**). Regarding Se-biofortified (+Se) plants, DS significantly reduced aerial part weight by 24% (**Table 1** and **Supplementary Table S3**). Therefore, although DS caused a reduction in maize development, the treatment with Se was able to reduce these adverse effects by at least 13%. Regarding irrigated plants (WW), Se-biofortification significantly increased the aerial part weight by 32% (**Table 1** and **Supplementary Table S3**). In terms of drought stressed (DS) plants, Se biofortification significantly increased the aerial part weight by 59%. Therefore, the beneficial effect of Se-treatment was higher on plants in water stressed conditions, resulting in an increase of the development of the treated plants by at least 27%, compared to the untreated controls (**Table 1** and **Supplementary Table S3**).

**Table 1:** Weight and total, inorganic [Se(IV)+Se(VI)] and organic (Total Se-Inorganic Se) Se concentrations in maize aerial parts and roots.

		Weight	Total Se	Inorganic Se	Organic Se
		(g plant^-1^)	(μg kg^-1^)	(μg kg^-1^)	(μg kg^-1^)
Aerial parts	WW-Se	4.09 b	328 b	218 b	100 c
	DS-Se	2.57 c	381 b	242 ab	139 b
	WW+Se	5.38 a	1,297 a	267 a	1,030 a
	DS+Se	4.09 b	1,321 a	275 a	1,046 a
Roots	WW-Se	Nd	883 b	785 b	98 b
	DS-Se	Nd	954 b	870 b	84 b
	WW+Se	Nd	4,520 a	2,630 a	1,890 a
	DS+Se	Nd	4,970 a	2,715 a	2,255 a

*The reported data are expressed on dry basis. Nd, not determined; WW, well watered; DS, drought stress. (WW-Se) normal irrigation without Se treatment, (DS**-**Se) drought stress without Se treatment, (WW+Se) normal irrigation with Se treatment and (DS+Se) Se under drought stress conditions. Data for each experiment were compared separately using Fisher’s LSD test (*P* < 0.05). Different letters indicate significant differences between treatments.*

Significant differences in total Se concentration were observed in aerial parts and roots of (+Se) and (-Se) maize plants, regardless of the stress conditions (**Table 1**). As concerns roots, Se bio-fortification enhanced the total Se concentrations by 5.1 and 5.2 times in irrigated and in drought stressed plants, respectively. Regarding aerial parts, Se bio-fortification enhanced total Se concentrations by 3.9 and 3.5 times in irrigated and in drought stressed plants, respectively. In the aerial part, the above increase in total Se appeared to be mainly due to the Se organic forms which increased by about ten times (**Table 1**) in (+Se) plants. On the contrary, in (+Se) plants the inorganic Se concentrations are similar to those of (-Se) plants. As concerns roots, the increase in total Se concentrations seems to be due both to inorganic and organic Se forms.

Considering the ‘aerial part/root’ ratios in relation to total Se concentration, Se-treated plants showed a decrease, both in irrigated and in drought stressed plants (**Table 2**).

**Table 2:** Ratio between Se in aerial parts and roots (DW), for total Se, organic Se (total Se less inorganic Se) and inorganic Se [sum Se(VI) and Se(IV)].

	Aerial part/root ratios		
	Total Se	Se organic	Se inorganic
WW-Se	0.37 a	1.31 a	0.28 a
DS-Se	0.40 a	1.39 a	0.28 a
WW+Se	0.29 b	0.56 b	0.10 b
DS+Se	0.26 b	0.45 b	0.10 b

*WW, well watered; DS, drought stress; DW, dry weight. (WW-Se) normal irrigation without Se treatment, (DS-Se) drought stress without Se treatment, (WW+Se) normal irrigation with Se treatment and (DS+Se) Se under drought stress conditions. Data for each experiment were compared separately using Fisher’s LSD test (*P* < 0.05). Different letters indicate significant differences between treatments.*

Selenium bio-fortification of irrigated plants caused significant increases of SeCys2 (448%), SeMeSeCys (698%), SeMet (383%), Se(IV) (449%), and Se(VI) (326%) in the roots, with respect to (-Se) (**Table 3**). Likewise, Se bio-fortification of drought stressed plants caused significant increases of SeCys2 (273%), SeMeSeCys (225%), and Se(VI) (379%) (**Table 3**). Regarding aerial parts, Se bio-fortification of irrigated plants caused significant increases of SeCys2, SeMeSeCys of 88.7 and 40.9% and significant decrease of SeMet of 29.5%. Likewise, in drought stressed plants, Se bio-fortification caused significant increases of SeCys2 (68.4%) and SeMeSeCys (34.8%), while SeMet significantly decreased (-36.4%). It is interesting to note that in irrigated plants, the Se organic forms which increased most with Se bio-fortification were SeMeSeCys and SeCys2, both in roots and in aerial parts; however, in drought stressed plants, Se bio-fortification mostly increased SeCys2, both in roots and in aerial parts (**Table 3**). As concerns Se inorganic forms, in irrigated plants, Se(IV) increased more than Se(VI) both in roots and in aerial parts; otherwise, in roots of S plants, Se(VI) was the form which increased the most. Concerning aerial parts, Se(IV) and Se(VI) showed similar increases (**Table 3**).

**Table 3:** Selenium speciation in maize aerial parts and roots.

		Se (μg kg^-1^) DW				
		SeCys_2_	MeSeCys	SeMet	Se(IV)	Se(VI)
roots	WW-Se	47.8 c	14.3 b	143.5 c	125.5 c	659.7 b
	DS-Se	80.9 b	38.8 c	196.7 b	254.1 b	616.4 c
	WW+Se	214.3 a	99.9 a	549.0 a	563.3 a	2,152.6 a
	DS+Se	221.1 a	87.2 b	243.0 b	293.4 b	2,337.5 a
aerial parts	WW-Se	95.3 c	2.2 b	25.4 b	60.1 b	158.6 b
	DS-Se	83.0 c	2.3 b	48.6 a	57.0 b	185.1 ab
	WW+Se	179.8 a	3.1 a	17.9 c	87.1 a	188.4 ab
	DS+Se	139.8 b	3.1 a	30.9 b	63.6 b	204.3 a

*The values reported are on dry basis. WW, well watered; DS, drought stress. (WW-Se) normal irrigation without Se treatment, (DS-Se) drought stress without Se treatment, (WW+Se) normal irrigation with Se treatment and (DS+Se) Se under drought stress conditions. Data for each experiment were compared separately using Fisher’s LSD test (*P* < 0.05). Different letters indicate significant differences between treatments.*

**FIGURE 1:**
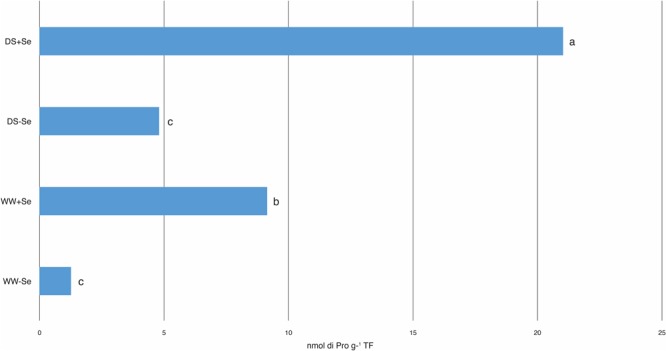
Proline (Pro) concentrations in aerial parts. WW, well watered; DS, drought stress. (WW–Se) normal irrigation without Se treatment, (DS–Se) drought stress without Se treatment, (WW+Se) normal irrigation with Se treatment and (DS+Se) Se under drought stress conditions. Data for each experiment were compared separately using Fisher’s LSD test (*P* < 0.05). Different letters indicate significant differences between treatments.

The production of proline in the aerial part of (+Se) plants, both irrigated and drought stressed, increased significantly compared to that of the (-Se) plants (**Figure 1**). Moreover, DS imposed on the (-Se) plants did not produce any increase in proline, confirming that this increase was due to Se (**Figure 1**).

**FIGURE 2:**
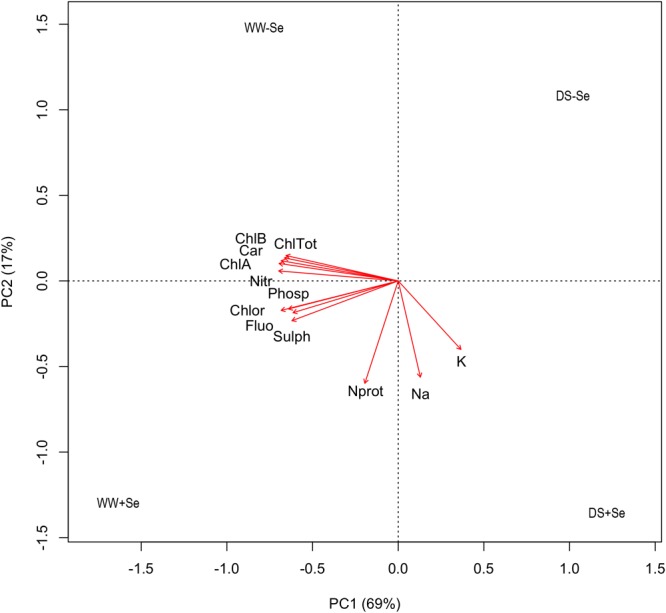
PCA on correlation matrix for the concentration of several compounds in aerial parts (DW). Sodium (Na); potassium (K); proteic nitrogen (Nprot); chlorophyll A (ChlA); chlorophyll B (ChlB); Total chlorophyll (TotChl); carotenoids (Car); nitrates (Nitr); fluorides (Fluo); sulphates (Sulph); phosphates (Phosph) and chlorides (Chlor); (WW–Se) normal irrigation without selenium treatment, (DS–Se) drought stress without Se treatment, (WW+Se) normal irrigation with Se treatment and (DS+Se) Se under drought stress conditions.

The whole set of results relating to maize aerial part samples was summarized by using a PCA (**Figure 2** and **Supplementary Table S4**) and the model thus obtained explains 86% of the total variance of data with two principal components (69 and 17%, respectively). The resulting biplot shows that the concentrations of Chlorophyll A (ChlA), chlorophyll B (ChlB), Total chlorophyll (TotChl), carotenoids (Car), nitrates (Nitr), fluorides (Fluo), sulphates (Sulph), phosphates (Phosph), and chlorides (Chlor) are mainly related to the positioning of observations along the first component (x-axis), while proteic nitrogen (Nprot), sodium (Na), and potassium (K) are mainly related to the positioning along the second component (y-axis). In this respect, DS-Se lies on the first quadrant (positive scores for both PCs) and it shows small values on all the observed components; WW-Se lies on the second quadrant (negative score for PC1 and positive for PC2) and was high in Chlorophyll A, B, carotenoids and nitrates and relatively low in all other compounds. WW+Se lies on the third quadrant (negative scores for both PCs) and shows high values of all compounds, while DS+Se lies on the fourth quadrant and it was low in all compounds, except Na, K, and Nprot. Indeed, water stress moves the observation along the x-axis and thus determines a decrease in all compounds except K, Na, and Nprot, which tend to increase in DS+Se plants. On the other hand, the treatment with Se moves the observations along the y-axis and treated plants tend to recover their concentrations in Na, K, and Nprot (irrigated and drought stressed) and in fluorides, sulfates, phosphates and chlorides (only irrigated plants).

### Extent and Pattern of DNA Methylation Under Control Conditions and Drought Stress/Selenium Treatment

A total of 653 clear and reproducible bands were amplified from 12 primer combinations (**Supplementary Table S2**) in plants grown either under normal irrigation or DS. Under control conditions (WW-Se), the total methylation of CCGG sites averaged 61.2%; this value decreased (-0.35% corresponding to 60.85%) in the presence of selenium (+Se), with the same irrigation conditions (**Supplementary Table S5**). In drought stressed samples (DS), DNA methylation level in the presence of Se was lower than in -Se samples (60.77% vs. 60.57%).

### Effect of Drought Stress and Presence of Se on the Level of Methylation in Maize

The 653 amplification products were classified as shown in **Table 4**. Patterns A–C represents monomorphic classes in which methylation pattern is the same following either the WW-Se or the WW+Se/ DS-Se/ DS+Se samples. Patterns D–H are indicative of cytosine demethylation, whereas possible cytosine methylation events induced in WW+Se/ DS-Se/ DS+Se are represented by patterns NO. Selenium treatment in normal irrigation (WW+Se) samples was found to induce 7 demethylation events and 5 methylation events. On the contrary, DS induced 8 and 3 methylation and 6 and 5 demethylation events in the absence (DS-Se) or presence (DS+Se) of Se treatment, respectively.

**Table 4:** Variations in DNA methylation pattern of *Z. mays* in different grown conditions: normal irrigation without (WW-Se) or with (WW+Se) selenium treatment and drought stress without (DS-Se) or with (DS+Se) selenium treatment.

		Methylation pattern				Number of bands		
	Class	WW-Se		WW+Se/DS-Se/ DS+Se		WW+Se	DS-Se	DS+Se
		HpaII	MspI	HpaII	MspI			
No changes	A	+	–	+	–	254	254	258
	B	–	+	–	+	105	101	105
	C	+	+	+	+	275	269	273
Demethylation	D	+	–	+	+	5	3	2
	E	–	+	+	+	0	0	2
	F	–	–	+	+	0	0	0
	G	–	+	+	–	0	0	0
	H	–	–	+	–	2	3	1
Methylation	I	+	+	+	–	2	4	2
	J	+	+	–	+	1	0	0
	K	+	+	–	–	0	0	0
	L	+	–	–	+	0	0	0
	M	+	–	–	–	2	4	1
Non informative	N	–	–	–	+	3	7	7
	O	–	+	–	–	4	8	2
Total						653	653	653

*+ and - indicate the presence and absence of band, respectively.*

### Statistical Analysis

Drought stress effect was significant (*P* < 0.05) for Non-, Total-, Full-Methylated bands (**Tables 5A–C**) and accounted for 28.7, 28.7, and 40.4% of the total sum of squares (SS), respectively, when WW-Se were compared with DS-Se and with DS+Se plants. Also, for the comparison between the same stresses and considering the comparison between Total- vs. Full- and Total- vs. Hemi- Methylated bands (**Tables 5D,E**), the stress effect was significant and accounted for 0.18 and 0.01% of total SS, respectively. However, the main source of variation for Total- vs. Full- Methylated and Total- vs. Hemi- Methylated bands was the effect Class (**Tables 5D,E**), that accounted for 99.6 and 99.9% of the total SS relative to main effects (Stress+Class+Rep). For Class Hemi-Methylated, the Types (i.e., I, II, III, and IV), the all possible comparison between Types and the other comparison between Classes (i.e., Full- vs. Hemi Methylated) only the effect Class was significant (data not shown).

**Table 5:** Analysis of variance for each different status of methylation (Not-, Total-, Full-, and Hemi- methylated bands) and for each pairwise combination of them considering all the pairwise combinations of stresses (drought stress and Se treatment).

(A) *Non methylated*				WW-Se vs. DS-Se		
Treatments	Df	SS	MS	*F* value	Pr(>*F*)	
Stress	1	33.333	33.333	11.364	0.01987	^∗^
Rep	5	68	13.6	4.637	0.05882	
Residuals	5	14.667	2.933	

**(B) *Total methylated***				**WW-Se vs. DS-Se**		
**Treatments**	**Df**	**SS**	**MS**	***F* value**	**Pr(>*F*)**	

Stress	1	33.333	33.333	11.364	0.01987	^∗^
Rep	5	68	13.6	4.637	0.05882	
Residuals	5	14.667	2.933	

**(C) *Full methylated***				**WW-Se vs. DS+Se**		
**Treatments**	**Df**	**SS**	**MS**	***F* value**	**Pr(>*F*)**	

Stress	1	65.333	65.333	11.395	0.01977	^∗^
Rep	5	67.667	13.533	2.361	0.18381	
Residuals	5	28.667	5.733	

**(D) *Total- and full-methylated***				**WW-Se vs. DS+Se**		
**Treatments**	**Df**	**SS**	**MS**	***F* value**	**Pr(>*F*)**	

Stress	1	131	131	23.316	0.00019	^∗∗∗^
Class	1	74148	74148	13.230.9	<2.00E-16	^∗∗∗^
Rep	5	112	22	3.997	0.01522	^∗^
Residuals	16	90	6	

**(E) *Total- and Hemi- Methylated***				**WW-Se vs. DS-Se**		
**Treatments**	**Df**	**SS**	**MS**	***F* value**	**Pr(>*F*)**	

Stress	1	54	54	65.787	0.02077	^∗^
Class	1	498240	498240	60699.31	<2.00E-16	^∗∗∗^
Rep	5	178	36	43.452	0.0109	^∗^
Residuals	16	131	8	

*A: not methylated bands, B: total methylated bands (i.e., the sum of full-methylated and hemi-methylated bands), C: full methylated bands, D: comparison between total and full methylated bands, and E: comparison between total and hemi methylated bands. (WW-Se) normal irrigation without selenium treatment, (DS-Se) drought stress without Se treatment, (WW+Se) normal irrigation with Se treatment and (DS+Se) Se under drought stress conditions. ^∗∗∗^*P* < 0.01; ^∗∗^*P* < 0.01; ^∗^*P* < 0.05. Table showed only significant *F* value for Stress treatment.*

### Sequencing and Bioinformatics Analysis of Methylated DNA Fragments

Nine differentially methylated DNA bands were excised from acrylamide gels, cloned and sequenced. The resulting sequences were blasted against the databases at NCBI, Uniprot and MaizeGDB websites. The sequences of three fragments were too short (under 75 nt) and other three resulted in no similarities. Three sequences showed a significant match (*E*-value lower that 0.001) and were significantly associated with *Z. mays* genes: (i) ZM_1 sequence (size 112 bp, accession number MG949279) showed a significant similarity (94.12% identity, *E*-value: 8.845e-7) with a maize gene encoding for chloroplast PSY1 (accession number Q6SMR0); (ii) ZM_2 sequence (size 174 bp, accession number MG949280) displayed was very like (96.43% identity, *E*-value: 6.776e-5) with a maize gene encoding for SDH (accession number DQ191049); (iii) ZM_3 sequence (size 106 bp, accession number MG949281) showed high similarity (97.37% identity, *E*-value 3.463e-11) with a maize gene encoding for an ADH1 (accession number AY691949).

## Discussion

The results gathered in this study show the effectiveness of Se bio-fortification in increasing water stress tolerance in maize by limiting the reduction of plant biomass due to water shortage conditions, as also reported by Nawaz et al. (2016). Hartikainen et al. (2000) reported that Se effects on plants depend on its concentration. At lower rates, Se stimulated growth of ryegrass seedlings in pot experiments, while at high doses it acted as pro-oxidant reducing yields and induced metabolic disturbances. This was also confirmed by Hawrylak-Nowak et al. (2015) in cucumber where root and aerial part biomass was inversely related to Se concentration.

Moreover, other studies showed that the exogenous application of Se increased the tolerance of plants to drought-induced oxidative damage by enhancing their antioxidant defense systems (Hasanuzzaman and Fujita, 2011; Proietti et al., 2013).

Regarding the effectiveness of the Se-biofortification method used in this study, the percentage increase of total Se concentration in the irrigated (WW) and drought stressed (DS) plants was of the same magnitude.

Our results show that Se bio-fortification (+Se) determined an increase in the Se amount in roots, which was greater than that observed in aerial parts (**Table 1**). Such behavior should be attributed to the selection of selenite as a fortifying agent, because of its greater persistence in the soil compared to selenate (Wang et al., 2013) and with little translocation properties to aerial parts (Arvy, 1993; De Souza et al., 1998; Zayed et al., 1998; Hopper and Parker, 1999; Terry et al., 2000; Ximénez-Embún et al., 2004; Li et al., 2008; Liu and Gu, 2009). As reported by Zhu et al. (2009), translocation of Se from roots to aerial parts depends on which Se species is supplied to the plant. In plants fed with selenate, Se is readily translocated to the aerial part. By contrast, in selenite-treated plants, most of Se stays in the roots and it is rapidly converted to organic forms; most of it, however, remains in inorganic and water-insoluble forms, as confirmed by our results (**Table 2**).

Data obtained from Se-speciation highlighted how the maize plant’s capacity to tolerate large tissue Se concentrations is primarily related to the ability to divert Se away from the accumulation of SeCys and SeMet, which might be incorporated into non-functional proteins through the synthesis of less toxic Se metabolites. Pilon-Smits and Quinn (2010) indicated that toxic SeCys can be methylated to form SeMeSeCys, a non-toxic free amino acid, by SeCys methyltransferase (SMT). Because, in contrast to Se-Cys and Se-Met, SeMeSeCys does not enter proteins and it can be safely accumulated to high levels in plant tissues, which in part explains the high tolerance of hyperaccumulators to Se. Furthermore, Zhang et al. (2006) reported, in *Arabidopsis thaliana* L., that tolerance to both selenate and selenite has been correlated with high SeCys_2_ concentrations.

These findings suggest resistance strategies to Se similar to those existing in Se hyperaccumulator species. Moreover, as SeMeSeCys is the form of Se which confers the best anticarcinogenic properties, this is advantageous from a human nutrition perspective (Wang et al., 2013).

The maize plants grown in Se-rich soils produced more proline than those grown in the untreated ones, regardless of water stress conditions. When exposed to stress factors, plants react by accumulating metabolites, such as amino acids, and proline is one of them. Several reports indicate that stressful environments may result in an overproduction of proline in the plants, which may contribute to stress tolerance, by maintaining cell turgor or osmotic balance, stabilizing membranes and thereby preventing electrolyte leakage; this brings concentrations of ROS within normal ranges, thus preventing oxidative burst in plants (Hayat et al., 2012). A similar increase of proline production has been reported by Khan et al. (2015), which studied the influence of Se in the protection of photosynthetic capacity of wheat (*Triticum aestivum*) against cadmium stress; they reported that an application of Se in the growth substrate of wheat seeds alleviated Cd-induced oxidative stress by increasing proline accumulation as a result of decreased activity of proline oxidase.

The analysis of the parameters regarding aerial parts highlights how Se-treated plants tend to recover their concentrations in Na, K, and Nprot (DS and WW plants) and in Fluo, Sulph, Phosph, and Chlor (only WW plants).

It is preferable not to hypothesize the causes of the increase of Na concentration in the analyzed plant material, because of its administration to the plant in the form of Na selenite. Interactions between Se and other elements, such as K, are also well-reported in literature. For example, Yao et al. (2013) documented that exogenous Se supply significantly enhanced the uptake of K in wheat, whereas, Pazurkiewicz-Kocot et al. (2003) noticed that Se supplementation increased K concentrations in maize grains. Indeed, Se is known to play important roles in inhibiting the production of ROS, which may be induced even by abiotic factors, such as DS. As the exposure to high levels of Se can possibly enhance membrane permeability, damage membrane integrity and produce high oxidative stress in treated plants, it was argued that the enhanced uptake of K might indicate that this element is involved in some mechanisms of Se tolerance within the plant (Feng et al., 2009).

Zahoor et al. (2017), reported that the increase of K in drought-stressed plants can sustain high nitrogen-metabolizing enzyme activities and contribute to osmotic adjustments in plants grown in soils under drought conditions.

The increase in Nprot concentrations, as a result of the Se-fortification, confirmed the results obtained by Ježek et al. (2011), when treating potato plants with foliar applications of sodium selenite. This increase is supposed to be another mechanism of resistance to an abiotic stress, induced by Se and implemented by the plant, which involves an increased intensity of amino acid metabolism.

As the increase in the concentration of Se in plants determines an increase in the concentration of K, which acts by limiting the damage due to DS inducing an increase in nitrogen metabolism, we can argue that Se itself induces tolerance toward DS.

The increase of the presence of anions, caused by Se-fortification of maize plants and which occurred only in good irrigation conditions. This may be related to the increased protein synthesis that may have induced an increase of the proteins that constitute the anion channel in the plasma membrane of cells, which are permeable to a range of physiological anions (Roberts, 2006), However, this hypothesis requires further confirmation.

In this paper, the MSAP technique was used in order to determine the level of DNA methylation in *Z. mays* under either water deficiency or Se treatment. The average of total relative cytosine methylation was 60.85% and was higher than that 26.15 and 32.15%, previously reported for maize leaves by Zhao et al. (2007) and Yang et al. (2011), respectively, but it was lower than 68.55% reported in the same species by Sun et al. (2015).

Positive effects of Se treatment were found to be associated with Se-mediated regulation of physiological and biochemical processes, such as increased chlorophyll and carotenoid concentrations and activation of antioxidant machinery in water stressed maize plants (Nawaz et al., 2016). Changes in DNA methylation could be considered a precise defensive mechanism to regulating the gene expression (Zhong et al., 2009).

In our study, the general methylation level of analyzed plants did not seem to be directly correlated with water stress and Se treatment, but it might be more influenced by genotype. In addition, Se supplementation seems to reduce the changes in DNA methylation caused by abiotic stress, probably due to its role in removing ROS produced by water stressed plant, as suggested by the low level of changes in methylation status in our samples. The protective role of Se against abiotic stress factors as high Cd concentration was also reported by Filek et al. (2008).

However, previous reports showed that environmental factors such as temperature, heavy metals and water stress as causing demethylation of genomic DNA (Chao et al., 2012). In fact, even if with small differences, methylation levels in water stressed plants without Se treatment were slightly higher than drought stressed plants with Se supplementation. These results seem to confirm that Se might reduce changes in methylation.

Among the few differentially methylated fragments, three were associated with known maize genes. In particular, ZM_1 displayed high similarity with a gene encoding for a chloroplast phytoene synthase (PSY1). Enzyme phytoene synthase catalyzes the conversion of two geranyl diphosphate in phytoene, which controls the flux of carotenoids. Carotenoids are necessary for photo protection and photosynthesis and they play an important role as a precursor to signaling molecules that influence plant development and biotic/abiotic stress responses. The gene family in maize and other grasses contains 3 paralogues. PSY1 is essential for maintaining leaf carotenoid content, particularly under heat stress growth conditions; the transcript level of PSY3 maize was regulated in response to abiotic stresses (Li et al., 2007), suggesting that PSY is involved in plant tolerance to abiotic stress (Han et al., 2008). In Pezzarossa et al. (2014), however, a reduction in β-carotene concentrations was also observed in tomato fortified with Se (1 mg Se L^-1^).

It had been reported that Se may down-regulate the expression of PSY, which was a key step of carotenoid biosynthesis in *Arabidopsis* (Sams et al., 2010). Moreover, Se was cited as down-regulating factor of some enzymes or genes of carotenoids synthesis in tomato (Pezzarossa et al., 2014).

ZM_2 displayed sequence homology with gene SDH. Plant SDH is the key enzyme in the sorbitol metabolism pathway (Nosarzewski and Archbold, 2007) and has been associated with resistance to abiotic stresses such as drought and salinity. SDH activity regulates the levels of polyols (Aguayo et al., 2013), which act as important osmolytes during DS and recovery processes.

ZM_3 displayed homology with gene encoding alcohol dehydrogenase 1 (ADH1). ADH activity may play a central role in biochemical adaptations to environmental stress. It is known to be induced during low oxygen stress, increasing in leaves and roots of plants exposed to flooding. In rice seeding, ADH1 and ADH2 are involved in flooding tolerance, but in maize and lettuce seedlings, the expression of genes is also responsive to wounding (Kato-Noguchi, 2001). The silencing of ADH in *N. benthamiana* and *N. tabacum* led to increased susceptibility of the plants to water deficit stress (Senthil-Kumar and Mysore, 2010).

As suggested by the methylation pattern of the fragments sequenced, Se did seem to bring back the methylation status. Indeed, in all the three bands the DS-Se caused changes in methylation status, while samples under DS treated by Se showed the same methylation pattern of controls.

## Conclusion

We observed that maize plants responded to Se treatment by activating some physiological and biochemical changes, in order to cope with DS. The Se-biofortification treatment proved effective in increasing Se concentrations in maize roots and aerial parts. Regarding the drought stressed maize plants grown in Se-rich substrates, the increase of proline, K concentrations and nitrogen metabolism in aerial parts seems to prove that Se-biofortification increased the plant’s resistance to water shortage conditions. The increase of SeMeSeCys and SeCys_2_ forms in roots and aerial parts of Se-treated plants, suggests the induction of resistance strategies similar to those existing in Se-hyperaccumulator species.

Moreover, as SeMeSeCys is the form of Se which confers the best anticarcinogenic properties, this is advantageous from an animal and human nutrition perspective.

In addition, the MSAP technique was used to assess the level of DNA methylation/demethylation in maize plants under DS coupled with Se treatment. The methylation level in DS+Se plants was, even if with small differences, lower than that of (DS-Se) ones. In addition, the level of methylation of DS+Se plants was more similar to that of WW-Se plants, suggesting that Se could have a role in preventing and/or counteracting changes in methylation status.

## Author Contributions

RD, DB, and EA conceived the experiments and followed all the trials. MF, CP, GB, and RD performed the chemical analysis (selenium speciation, ionic concentrations, proline concentrations, and determination of total nitrogen). MB performed the MSAP experiments. SC and VN performed the statistical analysis for the molecular part. AO performed the statistical analysis for the chemical part. MB, RD, and SC wrote the manuscript. MB, RD, SC, VN, GM, DB, AO, and EA revised the manuscript.

## Conflict of Interest Statement

The authors declare that the research was conducted in the absence of any commercial or financial relationships that could be construed as a potential conflict of interest.

## Footnotes

1 https://www.ncbi.nlm.nih.gov/

2 http://www.uniprot.org/

3 https://www.maizegdb.org/

## Supplementary Material

TABLE S1 Instrumental settings of the ICP-MS and HPLC.

TABLE S2 MSAP primer combination.

TABLE S3 Moisture of aerial parts and roots. Length of aerial parts.

TABLE S4 Principal component analysis, data.

TABLE S5 DNA methylation level in maize plants subjected to drought stress or not, treated or not with selenium.

## Abbreviations

ADH: alcohol dehydrogenase
ANOVA: analysis of variance
CAT: catalase
DAS: days after sowing
DS: drought stress
GF-AAS: graphite furnace atomic adsorption spectroscopy
GPX: glutathione peroxidase
HPLC: high performance liquid chromatography
LSD: least significant difference
MSAP: methylation sensitive amplified polymorphism
NCBI: National Center for Biotechnology Information
PCA: principal component analysis
PCR: C-reactive protein
PSY: phytoene synthase
PVC: polyvinyl chloride
ROS: reactive oxygen species
SDH: sorbitol dehydrogenase
SOD: superoxide dismutase
TOPO: topoisomerase
WW: well-watered
ZM: *Zea mays*

## References

[B1] Abul-Soud M. A. Abd-Elrahman S. H. (2016). Foliar selenium application to improve the tolerance of eggplant grown under salt stress conditions. *Int. J. Plant Soil Sci.* 9 1–10. 10.9734/IJPSS/2016/19992

[B2] Aguayo M. F. Ampuero D. Mandujano P. Parada R. Muñoz R. Gallart M. (2013). Sorbitol dehydrogenase is a cytosolic protein required for sorbitol metabolism in *Arabidopsis thaliana*. *Plant Sci.* 20 63–75. 10.1016/j.plantsci.2013.01.012 23498864

[B3] Akladious S. A. (2012). Influence of different soaking times with selenium on growth, metabolic activities of wheat seedlings under low temperature stress. *Afr. J. Biotechnol.* 11 14792–14804.

[B4] Arvy M. P. (1993). Selenate and selenite uptake and translocation in bean plants (*Phaseolus vulgaris*). *J. Exp. Bot.* 44 1083–1087. 10.1093/jxb/44.6.1083

[B5] Balal R. M. Shahid M. A. Javaid M. M. Iqbal Z. Anjum M. A. Garcia-Sanchez F. (2016). The role of selenium in amelioration of heat-induced oxidative damage in cucumber under high temperature stress. *Acta Physiol. Plant.* 38 1–14. 10.1007/s11738-016-2174-y

[B6] Bañuelos G. S. Lin Z. Q. Broadley M. (2017). *“Selenium Biofortification”, in Selenium in Plants*. Berlin: Springer 231–255. 10.1007/978-3-319-56249-0_14

[B7] Bocchini M. Bartucca M. Ciancaleoni S. Mimmo T. Cesco S. Pii Y. (2015). Iron deficiency in barley plants: phytosiderophore release, iron translocation and DNA methylation. *Front. Plant Sci.* 6:514. 10.3389/fpls.2015.00514 26217365 PMC4496560

[B8] Broadley M. R. Alcock J. Alford J. Cartwright P. Foot I. Fairweather-Tait S. J. (2010). Selenium biofortification of high-yielding winter wheat (*Triticum aestivum* L.) by liquid or granular Se fertilisation. *Plant Soil* 332 5–18. 10.1007/s11104-009-0234-4

[B9] Çakir R. (2004). Effect of water stress at different development stages on vegetative and reproductive growth of corn. *Field Crops Res.* 89 1–16. 10.1016/j.fcr.2004.01.005

[B10] Carter M. R. Gregorich E. G. (2007). *Soil Samplings and Methods of Analysis* 2nd Edn. Boca Raton, FL: CRC Press.

[B11] Cartes P. Jara A. A. Pinilla L. Rosas A. Mora M. L. (2010). Selenium improves the antioxidant ability against aluminium-induced oxidative stress in ryegrass roots. *Ann. Appl. Biol.* 156 297–307. 10.1111/j.1744-7348.2010.00387.x

[B12] Cataldi T. R. I. Margiotta G. Del Fiore A. Bufo S. A. (2003). Ionic content in plant extracts determined by ion chromatography with conductivity detection. *Phytochem. Anal.* 14 176–183. 10.1002/pca.700 12793466

[B13] Causevic A. Delaunay A. Ounnar S. Righezza M. Delmotte F. Brignolas F. (2005). DNA methylating and demethylating treatments modify phenotype and cell wall differentiation state in sugarbeet cell lines. *Plant Physiol. Biochem.* 43 681–691. 10.1016/j.plaphy.2005.05.011 16046142

[B14] Chao D. Y. Silva A. Baxter I. Huang Y. S. Nordborg M. Danku J. (2012). Genome-wide association studies identify heavy metal ATPase3 as the primary determinant of natural variation in leaf cadmium in *Arabidopsis thaliana*. *PLoS Genet.* 8:14. 10.1371/journal.pgen.1002923 22969436 PMC3435251

[B15] Chaoui A. Mazhoudi S. Ghorbal M. H. El Ferjani E. (1997). Cadmium and zinc induction of lipid peroxidation and effects on antioxidant enzyme activities in bean (*Phaseolus vulgaris* L.). *Plant Sci.* 127 139–147. 10.1016/S0168-9452(97)00115-5

[B16] Chilimba A. D. Young S. D. Black C. R. Meacham M. C. Lammel J. Broadley M. R. (2012). Agronomic biofortification of maize with selenium (Se) in Malawi. *Field Crops Res.* 125 118–128. 10.1016/j.fcr.2011.08.014

[B17] Cubadda F. Aureli F. Ciardullo S. D’Amato M. Raggi A. Acharya R. (2010). Changes in selenium speciation associated with increasing tissue concentration of selenium in wheat grain. *J. Agric. Food Chem.* 58 2295–2301. 10.1021/jf903004a 20102199

[B18] De Souza M. P. Pilon-Smits E. A. H. Lytle C. M. Hwang S. Tai J. Honma T. S. (1998). Rate-limiting steps in selenium assimilation and volatilization by Indian Mustard. *Plant Physiol.* 117 1487–1494. 10.1104/pp.117.4.1487 9701603 PMC34911

[B19] Dong J. Z. Wang Y. Wang S. H. Yin L. P. Xu G. J. Zheng C. (2013). Selenium increases chlorogenic acid, chlorophyll and carotenoids of Lycium chinense leaves. *J. Sci. Food Agric.* 93 310–315. 10.1002/jsfa.5758 22714393

[B20] Ducsay L. Ložek O. Varga L. (2009). Effect of selenium foliar application on its content in spring barley. *Agrochémia* 12 3–6.

[B21] Elkahoui S. Smaoui A. Zarrouk M. Ghrir R. Limam F. (2004). Salt-induced lipid changes in Catharanthus roseus cultured cell suspensions. *Phytochemistry* 65 1911–1917. 10.1016/j.phytochem.2004.06.021 15279997

[B22] EUROSTAT (2010). *Agricultural Statistics, Main Results 2008-2009*. Lugano: Pocketbooks.

[B23] Feng R. W. Wei C. Y. Tu S. Fengchang W. (2009). Effects of Se on the uptake of essential elements in *Pteris vittata* L. *Plant Soil* 325 123–132. 10.1007/s11104-009-9961-9

[B24] Filek M. Keskinen R. Hartikainen H. Szarejko I. Janiak A. Miszalski Z. (2008). The protective role of selenium in rape seedlings subjected to cadmium stress. *J. Plant Physiol.* 165 833–844. 10.1016/j.jplph.2007.06.006 17913288

[B25] Han H. Li Y. Zhou S. (2008). Overexpression of phytoene synthase gene from *Salicornia europaea* alters response to reactive oxygen species under salt stress in transgenic Arabidopsis. *Biotechnol. Lett.* 30 1501–1507. 10.1007/s10529-008-9705-6 18414806

[B26] Hanson B. Lindblom S. D. Loeffler M. L. Pilon-Smits E. A. (2004). Selenium protects plants from phloem-feeding aphids due to both deterrence and toxicity. *New Phytol.* 162 655–662. 10.1111/j.1469-8137.2004.01067.x 33873760

[B27] Hartikainen H. Xue T. Piironen V. (2000). Selenium as an anti-oxidant and pro-oxidant inryegrass. *Plant Soil* 225 193–200. 10.1023/A:1026512921026

[B28] Hasanuzzaman M. Fujita M. (2011). Selenium pretreatment upregulates the antioxidant defense and methylglyoxal detoxification system and confers enhanced tolerance to drought stress in rapeseed seedlings. *Biol. Trace Elem. Res.* 143 1758–1776. 10.1007/s12011-011-8998-9 21347652

[B29] Hawrylak-Nowak B. Matraszek R. Pogorzelek M. (2015). The dual effects of two inorganic selenium forms on the growth, selected physiological parameters and macronutrients accumulation in cucumber plants. *Acta Physiol. Plant* 37 1–13. 10.1007/s11738-015-1788-9

[B30] Hayat S. Hayat Q. Alyemeni M. N. Wani A. S. Pichtel J. Ahmad A. (2012). Role of proline under changing environments: a review. *Plant Signal. Behav.* 7 1456–1466. 10.4161/psb.21949 22951402 PMC3548871

[B31] Hopper J. L. Parker D. R. (1999). Plant availability of selenite and selenate as influenced by the competing ions phosphate and sulfate. *Plant Soil* 210 199–207. 10.1023/A:1004639906245

[B32] Isaac R. A. Johnson W. C. (1976). Determination of total nitrogen in plant tissue. *J. Assoc. Off. Anal. Chem.* 59 98–100. 6746471

[B33] Ježek P. Hlušek J. Lošák T. Jůzl M. Elzner P. Kráčmar S. (2011). Effect of foliar application of selenium on the content of selected amino acids in potato tubers (*Solanum tuberosum* L.). *Plant Soil Environ.* 57 315–320. 10.17221/57/2011-PSE

[B34] Kato-Noguchi H. (2001). Wounding stress induces alcohol dehydrogenase in maize and lettuce seedlings. *Plant Growth Regul.* 35 285–288. 10.1023/A:1014489922792

[B35] Khan M. I. R. Nazir F. Asgher M. Per T. S. Khan N. A. (2015). Selenium and sulfur influence ethylene formation and alleviate cadmium-induced oxidative stress by improving proline and glutathione production in wheat. *J. Plant Physiol.* 173 9–18. 10.1016/j.jplph.2014.09.011 25462073

[B36] Legendre P. Legendre L. (2012). *Numerical Ecology* 3rd Edn. Amsterdam, NL: Elsevier.

[B37] Li F. Murillo C. Wurtzel E. T. (2007). Maize Y9 encodes a product essential for 15-cis zetacarotene isomerization. *Plant Physiol.* 144 1181–1189. 10.1104/pp.107.098996 17434985 PMC1914175

[B38] Li H.-F. McGrath S. P. Zhao F.-J. (2008). Selenium uptake, translocation and speciation in wheat supplied with selenate or selenite. *New Phytol.* 178 92–102. 10.1111/j.1469-8137.2007.02343.x 18179602

[B39] Li M. Q. Hasan M. Li C. X. Ahammed G. J. Xia X. J. Shi K. (2016). Melatonin mediates selenium-induced tolerance to cadmium stress in tomato plants. *J. Pineal Res.* 61 291–302. 10.1111/jpi.12346 27264631

[B40] Lichtenthaler H. K. Wellburn A. R. (1983). Determinations of total carotenoids and chlorophylls a and b of leaf extracts in different solvents. *Biochem. Soc. Trans.* 11 591–592. 10.1042/bst0110591

[B41] Liu K. Gu Z. (2009). Selenium accumulation in different brown rice cultivars and its distribution in fractions. *J. Agric. Food Chem.* 57 695–700. 10.1021/jf802948k 19154168

[B42] Lukens L. N. Zhan S. (2007). The plant genome’s methylation status and response to stress: implications for plant improvement. *Curr. Opin. Plant Biol.* 10 317–322. 10.1016/j.pbi.2007.04.012 17468039

[B43] Marconi G. Pace R. Traini A. Raggi L. Lutts S. Chiusano M. (2013). Use of MSAP markers to analyse the effects of salt stress in DNA methylation in rapessed (*Brassica napus* var. oleifera). *PloS One* 8:e75597. 10.1371/journal.pone.0075597 24086583 PMC3781078

[B44] Nawaz F. Ahmad R. Ashraf M. Y. Waraich E. A. Khan S. Z. (2015). Effect of selenium foliar spray on physiological and biochemical processes and chemical constituents of wheat under drought stress. *Ecotoxicol. Environ. Saf.* 113 191–200. 10.1016/j.ecoenv.2014.12.003 25499052

[B45] Nawaz F. Naeem M. Ashraf M. Y. Tahir M. N. Zulfiqar B. Salahuddin M. (2016). Selenium supplementation affects physiological and biochemical processes to improve fodder yield and quality of maize (*Zea mays* L.) under water deficit conditions. *Front. Plant Sci.* 7:1438. 10.3389/fpls.2016.01438 27729917 PMC5037271

[B46] Nosarzewski M. Archbold D. D. (2007). Tissue-specific expression of sorbitol dehydrogenase in apple fruit during early development. *J. Exp. Bot.* 58 1863–1872. 10.1093/jxb/erm048 17404378

[B47] Palmerini C. A. Vedovelli A. Morelli A. Fini C. Floridi A. (2006). Analysis of acid-soluble hydroxy-proline, free proline and collagen-bound hydroxyproline in rat liver by high performance liquid chromatography with pre-column derivatization. *J. Liq. Chromatogr.* 8 1853–1868. 10.1080/01483918508074100

[B48] Pandey R. K. Maranville J. W. Admou A. (2000). Deficit irrigation and nitrogen effects on maize in a Sahelian environment: I. Grain yield and yield components. *Agric. Water Manae.* 46 1–13. 10.1016/S0378-3774(00)00073-1

[B49] Paszkowski J. Whitham S. A. (2001). Gene silencing and DNA methylation processes. *Curr. Opin. Plant Biol.* 4 123–129. 10.1016/S1369-5266(00)00147-3 11228434

[B50] Pazurkiewicz-Kocot K. Galas W. Kita A. (2003). The effect of selenium on the accumulation of some metals in *Zea mays* L. plants treated with indole-3-acetic acid. *Cell. Mol. Biol. Lett.* 8 97–103. 12655362

[B51] Peng H. Zhang J. (2009). Plant genomic DNA methylation in response to stresses: potential applications and challenges in plant breeding. *Prog. Nat. Sci.* 19 1037–1045. 10.1016/jpnsc.2008.10.014

[B52] Pezzarossa B. Rosellini I. Borghesi E. Tonutti P. Malorgio F. (2014). Effects of Se-enrichment on yield, fruit composition and ripening of tomato (*Solanum lycopersicum*) plants grown in hydroponics. *Sci. Hortic.* 165 106–110. 10.1016/j.scienta.2013.10.029

[B53] Pilon-Smits E. A. H. Quinn C. F. (2010). “Selenium metabolism in plants,” in *Cell Biology of Metals and Nutrients, Plant Cell Monographs* Vol. 17 eds Hell R. Mendel R.-R. (Berlin: Springer-Verlag) 225–241. 10.1007/978-3-642-10613-2_10

[B54] Proietti P. Nasini L. Del Buono D. D’Amato R. Tedeschini E. Businelli D. (2013). Selenium protects olive (*Olea europaea* L.) from drought stress. *Sci. Hortic.* 164 165–171. 10.1016/j.scienta.2013.09.034

[B55] Roberts S. K. (2006). Plasma membrane anion channels in higher plants and their putative functions in roots. *New Phytol.* 169 647–666. 10.1111/j.1469-8137.2006.01639.x 16441747

[B56] Rotruck J. T. Pope A. L. Ganther H. E. Swanson A. B. Hafeman D. G. Hoekstra W. (1973). Selenium: biochemical role as a component of glutathione peroxidase. *Science* 179 588–590. 10.1126/science.179.4073.588 4686466

[B57] Sams C. E. Panthee D. R. Charron C. S. Kopsell D. A. Yuan J. S. (2010). Selenium regulates gene expression for glucosinolate and carotenoid biosynthesis in Arabidopsis. *J. Am. Soc. Hortic. Sci.* 136 23–34.

[B58] Schwarz K. Foltz C. M. (1957). Selenium as an integral part of factor 3 against dietary necrotic liver degeneration. *J. Am. Chem. Soc.* 79 3292–3293. 10.1021/ja01569a087 10408880

[B59] Senthil-Kumar M. Mysore K. S. (2010). Assessing functional role of three water deficit stress-induced genes in nonhost disease resistance using virus-induced gene silencing in *Nicotiana benthamiana*. *Plant Signal. Behav.* 5 586–590. 10.4161/psb.11497 20436292 PMC7080461

[B60] Seppänen M. Turakainen M. Hartikainen H. (2003). Selenium effects on oxidative stress in potato. *Plant Sci.* 165 311–319. 10.1016/S0168-9452(03)00085-2

[B61] Sharpe J. (2009). Legislation. *J. Environ. Monit.* 11 1563–1569. 10.1039/B915684B

[B62] Sun L. F. Liu T. J. Shan X. H. Su S. Z. Li S. P. Yuan Y. P. (2015). Analysis of DNA cytosine methylation patterns in maize hybrids and their parents. *Biol. Plant.* 59 266–272. 10.1007/s10535-015-0490-5 25366740

[B63] Terry N. Zayed A. M. De Souza M. P. Tarun A. S. (2000). Selenium in higher plants. *Annu. Rev. Plant Biol.* 51 401–432. 10.1146/annurev.arplant.51.1.401 15012198

[B64] Teyssier E. Bernacchia G. Maury S. Kit A. H. Stammitti-Bert L. Rolin D. (2008). Tissue dependent variations of DNA methylation and endoreduplication levels during tomato fruit development and ripening. *Planta* 228 391–399. 10.1007/s00425-008-0743-z 18488247

[B65] The UniProt Consortium (2017). UniProt: the universal protein knowledgebase. *Nucleic Acids Res.* 45 D158–D169. 10.1093/nar/gkw1099 27899622 PMC5210571

[B66] US Environmental Protection Agency [EPA] (1996). *Method 3052B: Microwave Assisted Acid Digestion of Siliceous and Organically Based Matrices*. Washington, DC: USEPA.

[B67] Valkama E. Kivimäenpää M. Hartikainen H. Wulff A. (2003). The combined effects of enhanced UV-B radiation and selenium on growth, chlorophyll fluorescence and ultrastructure in strawberry (*Fragaria X ananassa*) and barley (*Hordeum vulgare*) treated in the field. *Agric. Forest Meteorol.* 120 267–278. 10.1016/j.agrformet.2003.08.021

[B68] Vitória A. P. Lea P. J. Azevedo R. A. (2001). Antioxidant enzymes responses to cadmium in radish tissues. *Phytochemistry* 57 701–710. 10.1016/S0031-9422(01)00130-3 11397437

[B69] Wang Y. D. Wang X. Wong Y. S. (2013). Generation of selenium-enriched rice with enhanced grain yield, selenium content and bioavailability through fertilisation with selenite. *Food Chem.* 141 2385–2393. 10.1016/j.foodchem.2013.05.095 23870972

[B70] Ximénez-Embún P. Alonso I. Madrid-Albarrán Y. Cámara C. (2004). Establishment of selenium uptake and species distribution in lupine, Indian mustard, and sunflower plants. *J. Agric. Food Chem.* 52 832–838. 10.1021/jf034835f 14969538

[B71] Yang W. Yu X. Liu B. (2011). Parental epigenetic difference in DNA methylation-level may play contrasting roles for different agronomic traits related to yield heterosis in maize. *Afr. J. Biotechnol.* 10 9253–9263. 10.5897/AJB11.298

[B72] Yao X. Chu J. Wang G. (2009). Effects of selenium on wheat seedlings under drought stress. *Biol. Trace Element Res.* 130 283–290. 10.1007/s12011-009-8328-7 19214397

[B73] Yao X. Jianzhou C. Xueli H. Binbin L. Jingmin L. Zhaowei Y. (2013). Effects of selenium on agronomical characters of winter wheat exposed to enhanced Ultra-Violet-B. *Ecotoxicol. Environ. Saf.* 92 320–326. 10.1016/j.ecoenv.2013.03.024 23597674

[B74] Zahoor R. Zhao W. Abid M. Dong H. Zhou Z. (2017). Potassium application regulates nitrogen metabolism and osmotic adjustment in cotton (*Gossypium hirsutum* L.) functional leaf under drought stress. *J. Plant Physiol* 215 30–38. 10.1016/j.jplph.2017.05.001 28527336

[B75] Zayed A. Lytle C. M. Terry N. (1998). Accumulation and volatilization of different chemical species of selenium by plants. *Planta* 206 284–292. 10.1007/s004250050402 18719961

[B76] Zhang L.-H. Abdel-Ghany S. E. Freeman J. L. Ackley A. R. Schiavon M. Pilon-Smits E. A. H. (2006). Investigation of selenium tolerance mechanisms in *Arabidopsis thaliana*. *Physiol. Plant* 128 212–223. 10.1111/j.1399-3054.2006.00739.x

[B77] Zhao X. Chai Y. Liu B. (2007). Epigenetic inheritance and variation of DNA methylation level and pattern in maize intra-specific hybrids. *Plant Sci.* 172 930–938. 10.1016/j.plantsci.2007.01.002

[B78] Zhong L. Xu Y. H. Wang J. B. (2009). DNA-methylation changes induced by salt stress in wheat *Triticum aestivum*. *Afr. J. Biotechnol.* 8 6201–6207. 10.1093/pcp/pcu059 24793752

[B79] Zhu Y. G. Pilon-Smits E. A. Zhao F. J. Williams P. N. Meharg A. A. (2009). Selenium in higher plants: understanding mechanisms for biofortification and phytoremediation. *Trends Plant Sci.* 436–442. 10.1016/j.tplants.2009.06.006 19665422

