# Supplementary material for: Soil Selenium (Se) Biofortification Changes the Physiological, Biochemical and Epigenetic Responses to Water Stress in Zea mays L. by Inducing a Higher Drought Tolerance
Source: Front Plant Sci. 2018 Mar 27;9:389. doi: 10.3389/fpls.2018.00389 (PMC5880925; doi:10.3389/fpls.2018.00389)
Supplement: TABLE S2 — MSAP primer combination. [file Table_2.DOCX]

**Supplementary Table S2**

| Adapter and Primer | *EcoR*I (5’-3’) | *Hpa*II */ Msp*I (5’-3’) |
| --- | --- | --- |
| Adapter 1 | CTCGTAGACTGCGTACC | GATCATGAGTCCTGCT |
| Adapter 2 | AATTGGTACGCAGTCTAC | CGAGCAGGACTCATGA |
| Pre-amplification primer | GACTGCGTACCAATTC+? | GATGAGTCTAGAACGG+? |
| Selective amplification primer | E+CCA | HM+AAT |
|  | E+CAA | HM+ACT |
|  | E+CAC | HM+ATC |
|  | E+CAG |  |
|  | E+CGG |  |

MSAP primer combinations
