# Supplementary material for: Soil Selenium (Se) Biofortification Changes the Physiological, Biochemical and Epigenetic Responses to Water Stress in Zea mays L. by Inducing a Higher Drought Tolerance
Source: Front Plant Sci. 2018 Mar 27;9:389. doi: 10.3389/fpls.2018.00389 (PMC5880925; doi:10.3389/fpls.2018.00389)
Supplement: TABLE S5 — DNA methylation level in maize plants subjected to drought stress or not, treated or not with selenium. [file Table_5.DOCX]

**Supplementary Table S5.** DNA methylation level in maize plants subjected to drought stress or not, treated or not with selenium.

| **MSAP band type** | **WW-Se** | **WW+Se** | **DS-Se** | **DS+Se** |
| --- | --- | --- | --- | --- |
| **I (not methylated)** | 253.33 ± 1.86 | 255.67 ± 1.51 | 256.17± 1.72 | 257.50± 3.39 |
| **II (hemimetilated)** | 111.33 ± 2.8 | 109.33 ± 2.94 | 109.83 ± 3.31 | 111.17 ± 1.72 |
| **III (full-methylated)** | 259.50 ± 1.05 | 256.83 ± 1.72 | 257.67 ± 2.5 | 256.67 ± 2.58 |
| **IV (full-methylated** | 28.84 ± 2.56 | 31.18 ± 3.92 | 29.34 ± 3.44 | 27.68 ± 2.73 |
| **Tot amplified bands** | 653 | 653 | 653 | 653 |
| **MSAP (%)** | 61.20 | 60.85 | 60.77 | 60.57 |

Data are means ± SD (n=6).

(WW-Se) normal irrigation without selenium treatment, (DS-Se) drought stress without Se treatment, (WW+Se) normal irrigation with Se treatment and (DS+Se) Se under drought stress conditions.
